# Regional and cellular organization of the autism-associated protein UBE3A/E6AP and its antisense transcript in the brain of the developing rhesus monkey

**DOI:** 10.3389/fnana.2024.1410791

**Published:** 2024-05-30

**Authors:** Chavely Gonzalez Ramirez, Sarah G. Salvador, Ridthi Kartik Rekha Patel, Sarah Clark, Noah W. Miller, Lucas M. James, Nicholas W. Ringelberg, Jeremy M. Simon, Jeffrey Bennett, David G. Amaral, Alain C. Burette, Benjamin D. Philpot

**Affiliations:** ^1^Neuroscience Center, University of North Carolina at Chapel Hill, Chapel Hill, NC, United States; ^2^Department of Cell Biology and Physiology, University of North Carolina at Chapel Hill, Chapel Hill, NC, United States; ^3^Department of Biostatistics, Harvard T.H. Chan School of Public Health, Boston, MA, United States; ^4^Department of Data Science, Dana-Farber Cancer Institute, Boston, MA, United States; ^5^Department of Psychiatry and Behavioral Sciences, MIND Institute, Davis, CA, United States; ^6^California National Primate Research Center, University of California, Davis, CA, United States; ^7^Carolina Institute for Developmental Disabilities, University of North Carolina at Chapel Hill, Chapel Hill, NC, United States

**Keywords:** Angelman syndrome, Dup15q syndrome, E6-AP, *UBE3A-ATS*, rhesus macaque, hippocampus

## Abstract

Angelman syndrome (AS) is a neurogenetic disorder caused by mutations or deletions in the maternally-inherited *UBE3A* allele, leading to a loss of UBE3A protein expression in neurons. The paternally-inherited *UBE3A* allele is epigenetically silenced in neurons during development by a noncoding transcript (*UBE3A-ATS*). The absence of neuronal UBE3A results in severe neurological symptoms, including speech and language impairments, intellectual disability, and seizures. While no cure exists, therapies aiming to restore UBE3A function—either by gene addition or by targeting *UBE3A-ATS*—are under development. Progress in developing these treatments relies heavily on inferences drawn from mouse studies about the function of UBE3A in the human brain. To aid translational efforts and to gain an understanding of UBE3A and *UBE3A-ATS* biology with greater relevance to human neurodevelopmental contexts, we investigated UBE3A and *UBE3A-ATS* expression in the developing brain of the rhesus macaque, a species that exhibits complex social behaviors, resembling aspects of human behavior to a greater degree than mice. Combining immunohistochemistry and *in situ* hybridization, we mapped UBE3A and *UBE3A-ATS* regional and cellular expression in normal prenatal, neonatal, and adolescent rhesus macaque brains. We show that key hallmarks of UBE3A biology, well-known in rodents, are also present in macaques, and suggest paternal *UBE3A* silencing in neurons—but not glial cells—in the macaque brain, with onset between gestational day 48 and 100. These findings support proposals that early-life, perhaps even prenatal, intervention is optimal for overcoming the maternal allele loss of *UBE3A* linked to AS.

## 1 Introduction

Maintaining the integrity of the proteome through effective protein turnover is essential for the proper functioning of all cells (Hinkson and Elias, 2011; Ross et al., 2021). Proteins that are unnecessary for current functions, misfolded, or damaged (due to oxidative stress, disease, or aging) must undergo degradation, with their amino acids recycled. This process is especially critical for cells with extended lifespans and intricate morphologies, such as neurons (Hanus and Schuman, 2013; Morozov and Karpov, 2018; Patrick et al., 2023). Beyond the shared processes common to all cells, protein degradation serves unique functions in neurons, such as shaping synapse formation and pruning during development and regulating excitatory and inhibitory transmission in the mature brain (Jarome and Helmstetter, 2013; Turker et al., 2021).

**Figure 1:**
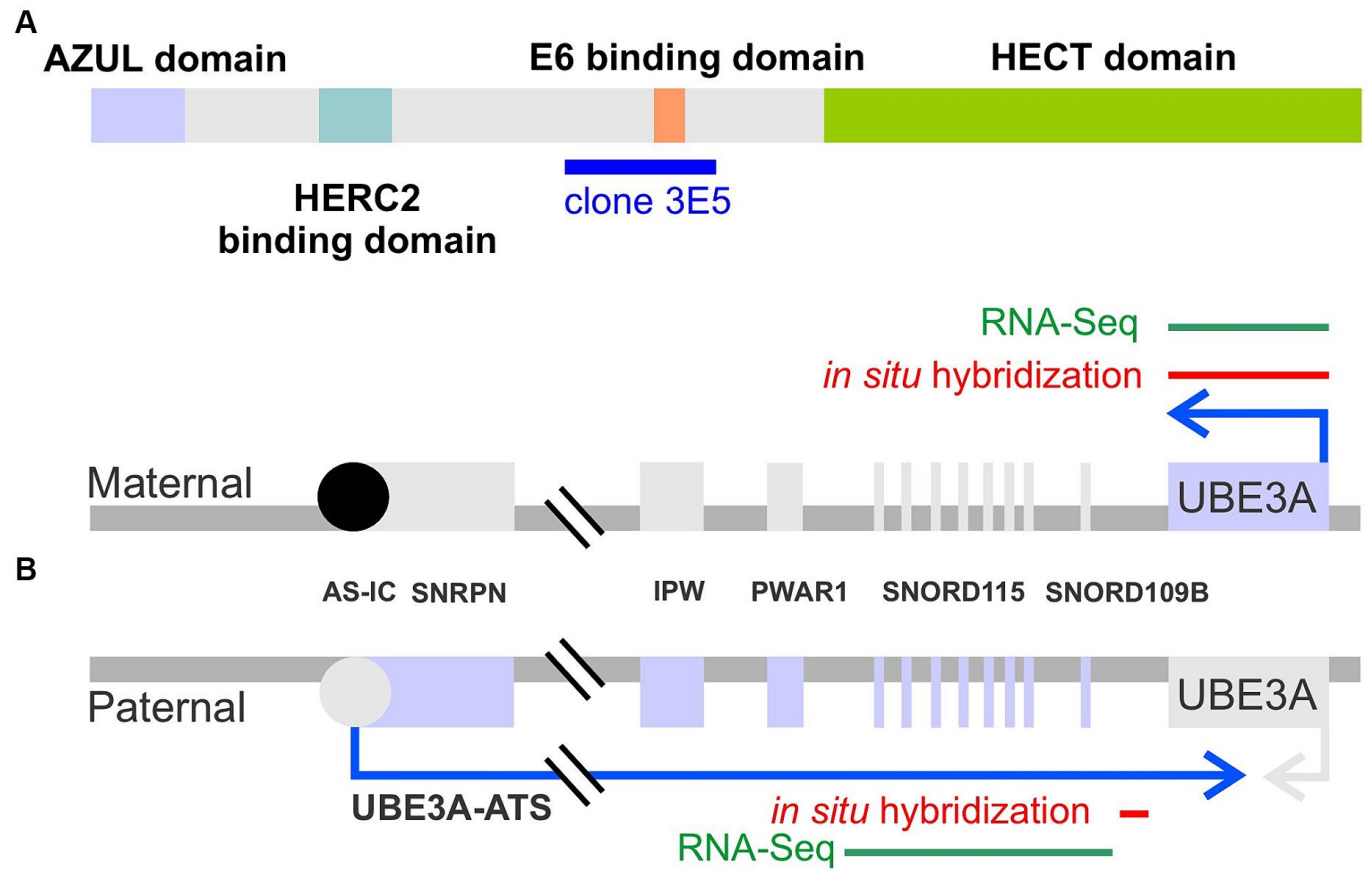
Schematic representation of UBE3A protein and *UBE3A* maternal and paternal alleles. **(A)** UBE3A protein schematic depicting the known functional domains and the epitope, clone 3E5, used to generate the antibody employed in this study. **(B)** *UBE3A* alleles depicting the location of the *in situ* hybridization probes used in this study, and sequence targets used for human RNA sequencing mining.

The ubiquitin-proteasome system is the primary mechanism responsible for the targeted degradation of aberrant and short-lived regulatory proteins (Nath and Shadan, 2009). This system involves a cascade of reactions successively catalyzed by three families of enzymes: ubiquitin-activating E1, ubiquitin-conjugating E2, and ubiquitin-ligase E3 enzymes. E3 ligases are noteworthy as they can directly bind to substrates and typically provide specificity for the substrate protein. The significance of E3 ligases is underscored by the many cellular processes they regulate and, correspondingly, the numerous diseases, including several neurological disorders, associated with their loss of function or mistargeting (George et al., 2018; Kawabe and Stegmuller, 2021). Two primary classes of E3 enzymes exist: HECT (homologous to E6-AP carboxyl-terminus) domain E3s and RING finger E3s. Due to its association with several neurological disorders, the ubiquitin protein ligase E3A (UBE3A, OMIM# 601623), the first E3 discovered in the HECT class (Figure 1A), holds particular significance in the central nervous system.

In the central nervous system, *UBE3A*, the gene that encodes UBE3A protein, undergoes imprinting, wherein the paternally-inherited allele is epigenetically silenced in neurons during development (Figure 1B) (Rougeulle et al., 1997; Judson et al., 2014). Mechanistically, paternal *UBE3A* silencing is disrupted by the transcription of an opposing long, non-coding RNA commonly referred to as *UBE3A-ATS* (Rougeulle et al., 1998; Hsiao et al., 2019). Consequently, only the maternal copy of *UBE3A* is expressed in neurons. Mutation or deletions of the maternally-inherited *UBE3A* allele leads to Angelman syndrome (AS, OMIM # 105830), a rare neurodevelopmental disorder characterized by developmental delay, severe intellectual disability, absence of speech, seizures, and ataxia (Buiting et al., 2016). Conversely, gain-of-function mutations in *UBE3A,* or the overexpression of *UBE3A* caused by extra copies of the 15q11-q13 chromosomal region in which *UBE3A* resides, are associated with autism spectrum disorder (Dup15q syndrome) (Hecht, 1975; Christian et al., 2008; Glessner et al., 2009; Xing et al., 2023).

Despite the identification of various putative targets of UBE3A protein (also known as E6-AP) (Sell and Margolis, 2015), the mechanisms governing the downstream effects of UBE3A gain or loss of function in neurodevelopmental disorders remain poorly understood, and effective treatments are lacking. As a result, current treatments for AS mainly focus on symptom management, utilizing anti-seizure medications and physical therapy approaches (Duis et al., 2022). Recent research by our lab and others has focused on reinstating the UBE3A protein, which can be achieved by reintroducing the *UBE3A* gene (Judson et al., 2021) or suppressing the *UBE3A-ATS* to reinstate expression of the paternal *UBE3A* allele (Huang et al., 2011; Meng et al., 2015; Wolter et al., 2020). While restoring UBE3A protein function holds potential therapeutic benefits, several obstacles must be addressed. Comprehensive understanding of UBE3A expression in a region- and cell-type-specific manner, both during early developmental stages and throughout adulthood, is *a sine qua non* for developing and evaluating potential therapeutic safety and effectiveness.

While human induced pluripotent stem cells provide some insights into UBE3A expression in humans (Chamberlain et al., 2010; Sirois et al., 2020), our knowledge of UBE3A expression within the intact human brain remains limited and has primarily taken advantage of western blotting (Williams et al., 2010; Daily et al., 2012). We previously used immunohistochemistry to explore UBE3A distribution in the human brain, but our focus was constrained to the adult temporal cortex due to the scarcity of high-quality human tissue samples (Burette et al., 2018). The assumption of ubiquitous UBE3A expression in the human brain and the broader idea of conserved UBE3A biology between humans and rodents stems primarily from research conducted on mice, but direct evidence is lacking. While there are optimistic grounds to believe in the validity of this assumption, caution is warranted.

This study aims to fill existing gaps in knowledge by exploring the temporal and spatial expression patterns of UBE3A and *UBE3A-ATS* in the developing and adult brains of macaque monkeys. Macaques are a compelling model for the study of neurodevelopmental disorders (Aida and Feng, 2020). Macaque and humans share numerous brain regions, circuits, and cell types critical for higher cognitive functions. Additionally, macaques exhibit advanced cognitive and social communication abilities, including face recognition, eye gazing, vocalizations, and complex behavioral patterns that are challenging to model in rodents.

## 2 Methods

### 2.1 Animals

Tissue from 11 macaque monkeys (*Macaca mulatta*) were used in the present study at the following ages: gestational day (GD) 48, male; GD 100, sex unknown; GD 151, male; 14 days, female; 14 days, male; 1 month and 3 days, male; 1 month, male; 2 months and 24 days, male; 3 months and 3 days, male; 5 years 4 months and 14 days, male (Table 1). The average pregnancy of a rhesus monkey is approximately 166 days. These sections were obtained from the tissue repository at the Amaral laboratory, and no animals were killed specifically for this project. Tissue from these animals were used in several other studies (e.g., Jabes et al., 2010).

**Table 1:** Age and sex of macaque specimens.

Date	Age	Sex
1/19/2007	48 days gestation	Male
3/16/2007	100 days gestation	Unknown
3/27/2007	151 days gestation	Male
5/18/2000	2 weeks	Female
6/4/1999	2 weeks	Male
4/19/1999	1 month 3 days	Male
6/24/1999	1 month	Male
6/28/1999	2 month 24 days	Male
7/28/1999	3 month 3 days	Male
6/22/2004	5 years 4 months	Male

### 2.2 Perfusion and sectioning

Animals were deeply anesthetized with pentobarbital and transcardially perfused. Perfusion of the postnatal cases followed a standard “modified immuno-perfusion” which entails transcardially perfusion with phosphate buffered 1% paraformaldehyde at 4°C at a rate of 250 mL/min for 2 min followed by 4% paraformaldehyde at the same rate and temperature for 10 min and continuing with 4% paraformaldehyde at a rate of 100 mL/min for 50 min. After which the brain was extracted and postfixed for 6 h in 4% paraformaldehyde. After postfixing, the brain was cryoprotected with 10% glycerol and 2% DMSO overnight followed by 20% glycerol with 2% DMSO for approximately 72 h at which time it was frozen in isopentane chilled with a dry ice and ethanol bath. Perfusions of the fetal cases were modified primarily by using a slower flow rate and shorter duration of fixative (e.g., 4 mL/min for 25 min for the GD 48 case; 25 mL/min for the GD 100 case; 75 mL/min for the GD 151 case).

All fetal brains were sectioned on a freezing sledge microtome without blocking using cryogel to fix the brain to the stage and powdered dry ice to keep the specimen frozen. All other brains were blocked at approximately -5 mm AP and affixed to the microtome stage using OCT and phosphate buffer. Sectioning scheme was the same for all brains. Sections were cut at 30 μm in a 1:8 series with series 1–3 and 5–8 collected in a cryopreservative solution while series 4 was collected into 10% buffered formalin to be fixed for additional 4 weeks before mounting for Nissl stain with thionin. Cryopreserved sections were stored at −80°C (Rosene et al., 1986). We utilized the tissue preservation method of Rosene et al. (1986), specifically designed for the long-term storage of valuable nonhuman primate tissue. The Amaral laboratory and many other primate and human neuroanatomy laboratories have been using this strategy for several decades. The universal finding is that tissue stored at −80 degrees Celsius suffers little or no degradation. Immunohistochemical staining separated by 10 or more years show virtually identical outcomes. This process has enabled all available tissue to be used and reduced the number of nonhuman primates that needed to be sacrificed for neuroscience research.

### 2.3 Antibodies

To label UBE3A, we selected the mouse monoclonal antibody 3E5 (Sigma-Aldrich Cat# SAB1404508, RRID:AB_10740376). This widely used antibody offers several advantages, including extensive validation (Judson et al., 2014; Burette et al., 2017; Sen et al., 2021). As a monoclonal antibody, it offers the advantage of being producible in virtually unlimited quantities with consistent properties, ensuring consistency and reproducibility across experiments over extended periods. The antibody was developed against a peptide sequence present in all three isoforms of mouse UBE3A (amino acids 315–415 for isoforms 1 & 3, and 336–436 for isoform 2) – as detailed in Figure 1A. Notably, this immunogen sequence exhibits high (98%) identity (99 out of 101 amino acids) with the macaque UBE3A protein.

To label SOX9, we used a polyclonal goat IgG (R and D Systems Cat# AF3075, RRID:AB_2194160) developed using a recombinant human SOX9 (Met1-Lys151). To label Oligodendrocyte Transcription Factor 2 (Olig2), we used a rabbit polyclonal anti-Olig2 antibody (Millipore Cat# AB9610, RRID:AB_570666) developed using a recombinant mouse Olig-2. This antibody specifically labels oligodendrocyte lineage cells (McTigue and Tripathi, 2008), which is consistent with our observations (Judson et al., 2014). To label NeuN antigen, we use a guinea pig polyclonal antibody (Millipore Cat# ABN90, RRID:AB_11205592) developed using GST-tagged recombinant fragment corresponding to the first 97 amino acids of mouse NeuN.

### 2.4 Immunohistochemistry

For immunoperoxidase microscopy, coronal sections were divided into two halves. One was Nissl stained to provide anatomical context, while the other was stained for UBE3A. Briefly, free-floating sections were first treated for 30 min with 3% H_2_O_2_ in phosphate-buffered saline (PBS, 0.1 M, pH 7.4). After preincubation in 1% BSA in PBS, sections were incubated overnight in UBE3A primary antibody (1:500) on a shaker at room temperature. Sections were then incubated for 3 h in biotinylated secondary antibody (1:400; Jackson ImmunoResearch, West Grove, PA) and for 1 h in ExtrAvidin-peroxidase complex (1:5,000; Sigma, St. Louis, MO); peroxidase was histochemically visualized with nickel-intensified diaminobenzidine. Processed sections were mounted on gelatin-coated slides, air-dried, and cleared with xylene before being coverslipped with D.P.X. mountant (BDH Chemicals, Poole, England). Sections were examined with a Nikon Eclipse Ti2.

For immunofluorescence, antigenic sites were visualized with donkey IgG conjugated to Alexa Fluor dyes. DAPI was used for counterstaining to reveal nuclei. Sections were examined with a Leica SP8X Falcon microscope. For analysis, images were imported in Qupath v0.4 (Bankhead et al., 2017). Cell detection was performed using a QuPath-implementation of the StarDist algorithm (Schmidt et al., 2018).

### 2.5 Hybridization chain reaction fluorescent *in situ* hybridization

Hybridization chain reaction fluorescent *in situ* hybridization (HCR-FISH) labeling kits were purchased from Molecular Technologies (Molecular Technologies, Pasadena, California), and experiments were performed following the manufacturer’s instructions. Probe sets used in this study include *UBE3A* (B3 initiator) and *UBE3A-ATS* (Figure 1B, B1), SOX9 (B2), VGLUT1 (B2), and VGAT (B4), and were detected using appropriate amplifier hairpins labeled with Alexa Fluor 488, 546, or 647. The location of the *UBE3A* and *UBE3A-ATS* probes are indicated in Figure 1B. Sections were examined with Leica SP8X Falcon and with Leica STELLARIS 8 FALCON microscopes.

### 2.6 Public RNA-seq data mining

Summarized human RNA-seq data were obtained from BrainSpan^1^ and filtered for exons of *UBE3A* or exons from the neuron-specific portion of the *UBE3A-ATS* (chr15: 25400000–25550000; hg19/GRCh37) as described by Hsiao et al. (2019) (Figure 1B). Expression data were categorized according to brain region and developmental stage, as previously described (Judson et al., 2021). Brain regions were grouped as prefrontal cortex (DFC, DFC, VFC, MFC, OFC), hippocampus (HIP), striatum (STR), thalamus (DTH, MD), and cerebellum (CB, CBC). Developmental periods were grouped as 1st trimester (8–13 postconceptional weeks PCW), 2nd trimester (16–25 PCW), 3rd trimester (26–37 PCW), early postnatal (4 months–2 years), early childhood (3–11 years), and adolescent (13–19 years). Expression of *UBE3A* and *UBE3A-ATS* are presented as reads per kilobase per million reads (RPKM) and plotted in R v4.1 using ggplot2 v3.3.6.

## 3 Results

**Figure 2:**
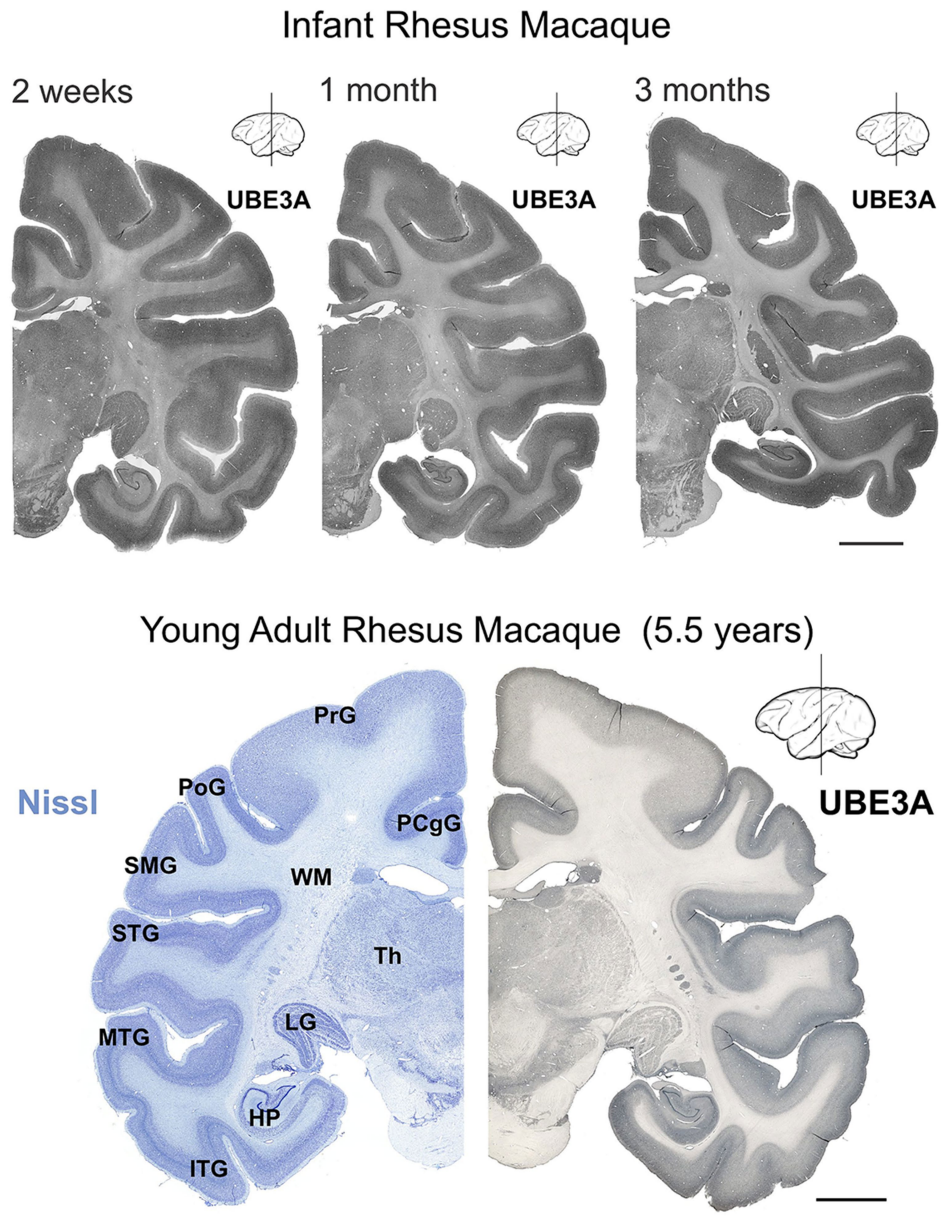
UBE3A expression in the infant and young adult rhesus macaque brain. UBE3A staining in coronal sections from 2 weeks-, 1 month-, 3 months-old, and 5.5 years-old rhesus macaque brain. Scale bar: 0.5 cm. HP, hippocampal formation (dentate gyrus, hippocampus, subiculum, presubiculum, parasubiculum); ITG, inferior temporal gyrus; LG, lateral geniculate nucleus; MTG, middle temporal gyrus; PCgG, posterior cingulate gyrus; PoG, postcentral gyrus; PrG, precentral gyrus; SMG, supramarginal gyrus; STG, superior temporal gyrus; Th, thalamus; WM, white matter.

**Figure 3 fig3:**
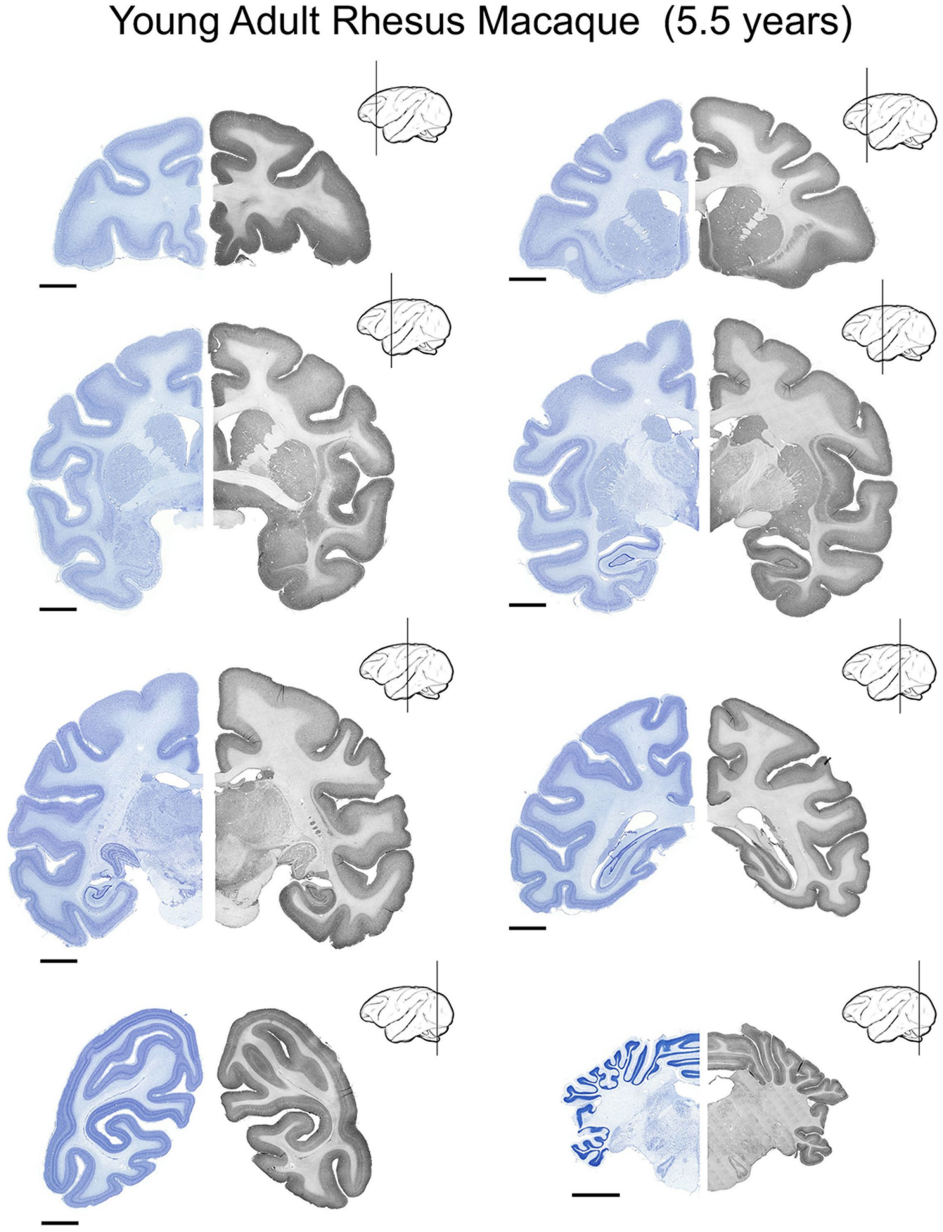
UBE3A expression in the young adult (5.5 years-old) rhesus macaque brain. UBE3A expression in 8 coronal sections across the young adult rhesus macaque brain. Each coronal section was divided into two halves. The Nissl image on the left side is from an adjacent series to the section shown on the right. The sections on the right underwent immunoperoxidase staining for UBE3A. Scale bars: 0.5 cm.

We first mapped UBE3A protein expression in the postnatal macaque brain using immunohistochemistry (Figures 2–4). The regional distribution of UBE3A in the infant macaque brain (2 weeks, 1 month, and 3 months of age) closely resembled that of the adolescent brain (5.5-year-old, Figure 2). Immunoperoxidase-reacted sections exhibited staining for UBE3A throughout the post-natal macaque brain, concentrating in the grey matter (Figures 2, 3). The hippocampus, cerebellum, and neocortex exhibited the highest UBE3A staining. The striatum, thalamus, and brainstem displayed comparatively weaker staining. The white matter exhibited the least intense staining.

**Figure 4:**
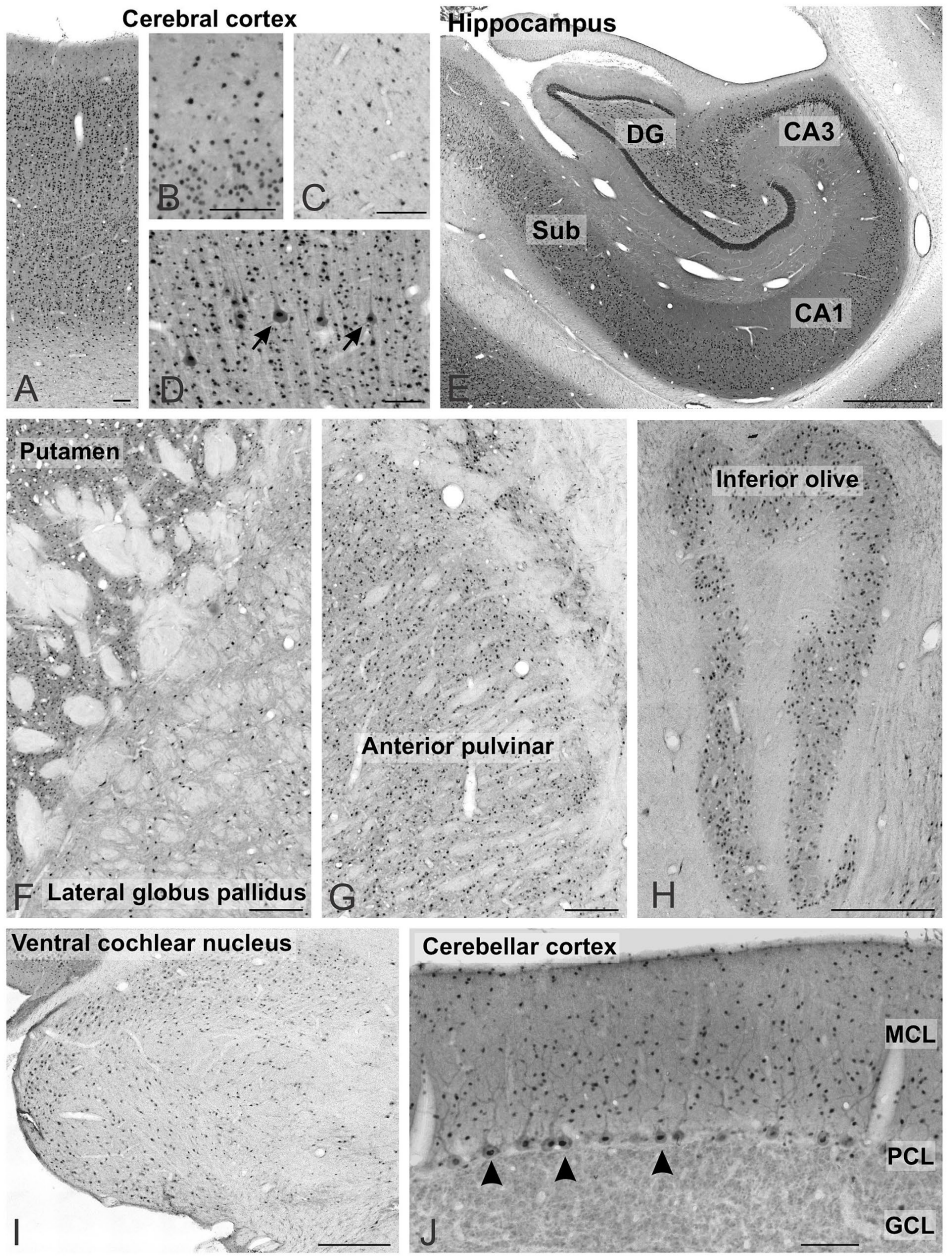
UBE3A expression across multiple regions of the one-month-old macaque brain. **(A–D)** Cerebral cortex, arrows point to pyramidal cells. **(E)** Hippocampus. **(F)** Striatum and pallidum. **(G)** Thalamus. **(H)** Inferior olive, **(I)** Ventral cochlear nucleus. **(J)** Cerebellar cortex, arrows point to Purkinje cells. Scale bars: **A–D**: 100 μm, **E–G**: 1 mm, **J,I**: 500 μm; **H**: 100 μm. CA1, CA3 fields of the hippocampus; DG, dentate gyrus; GCL, granule cell layer; MCL, molecular cell layer; PCL, Purkinje cell layer; Sub, subiculum.

UBE3A was found in all layers of the cerebral cortex (Figure 4A). Staining was weaker in layer 1 (Figure 4B) and the white matter (Figure 4C), reflecting lower cell density. At the subcellular level, the staining concentrated in nuclei, with comparatively weaker staining detected in somata and dendrites (Figure 4D). In the hippocampus, UBE3A staining ranged from moderate to intense in layers abundant in somata, encompassing the pyramidal cell layer of the hippocampus and the granule cell layer of the dentate gyrus (Figures 2, 3, 4E). Staining was comparatively lower in the paucicellular stratum radiatum and oriens. The subcellular distribution mirrored that of the neocortex, with prominent nuclear staining and weaker staining in the neuropil. In the basal ganglia, strongly stained nuclei were dispersed throughout the gray matter, while the fascicles of myelinated fibers exhibited minimal staining (Figure 4F). Positive nuclei were also present in the diencephalon (Figure 4G) and rhombencephalon (Figures 4H–I). In the cerebellar cortex, UBE3A staining concentrated in the nuclei of Purkinje cells, and in small nuclei scattered in the molecular layer (Figure 4J). In contrast, staining was lower in the nuclei of granule cells.

**Figure 5:**
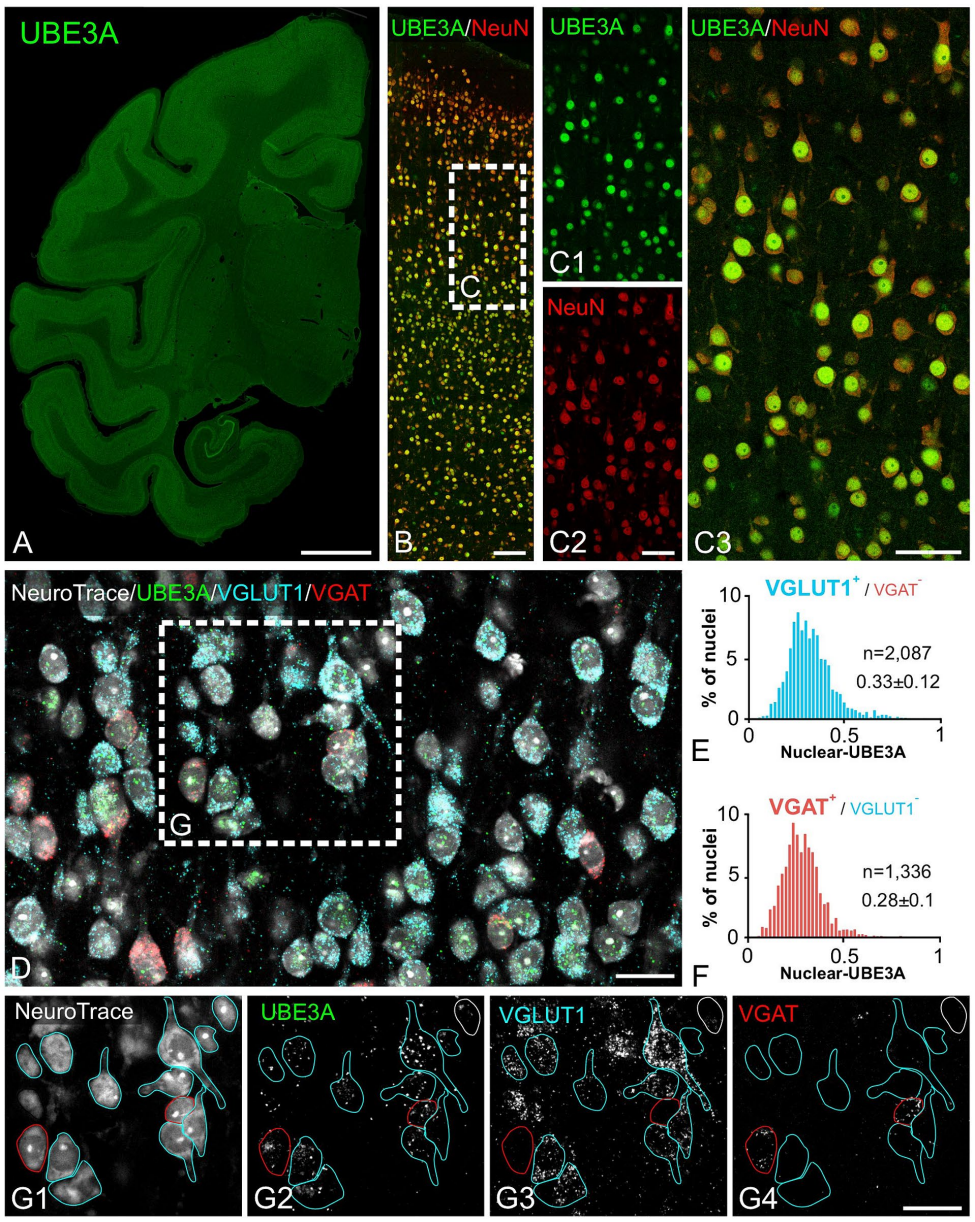
Both excitatory and inhibitory neurons exhibit strong UBE3A expression in the one-month-old macaque brain. **(A–C)** Double immunofluorescence labeling for UBE3A (green) and the neuronal marker NeuN (red). **(A)** UBE3A immunofluorescence labeling in a coronal section. **(B)** Double immunofluorescence staining for UBE3A (green) and NeuN (red) in the somatosensory cortex. **(C)** Enlargement of the boxed area from **B**; nearly all NeuN-positive cells (red) are also positive for UBE3A (green). **(D)** Triplex detection of UBE3A (green), the glutamatergic neuron marker VGLUT1 (blue), and the GABAergic marker VGAT (red) using HCR RNA fluorescence *in situ* hybridization. **(E)** The histogram plot shows nuclear UBE3A fluorescence profiles in excitatory neurons (VGLUT1 positive, but negative for VGAT). **(F)** The histogram plot shows nuclear UBE3A fluorescence profiles in inhibitory neurons (VGAT positive, but negative for VGLUT1). **(G)** Enlargement of the boxed area from **D**. Images are from the somatosensory cortex. Scale bars: **A**: 0.5 cm, **B**: 100 μm; **C**: 50 μm; **D,E**: 25 μm.

**Figure 6:**
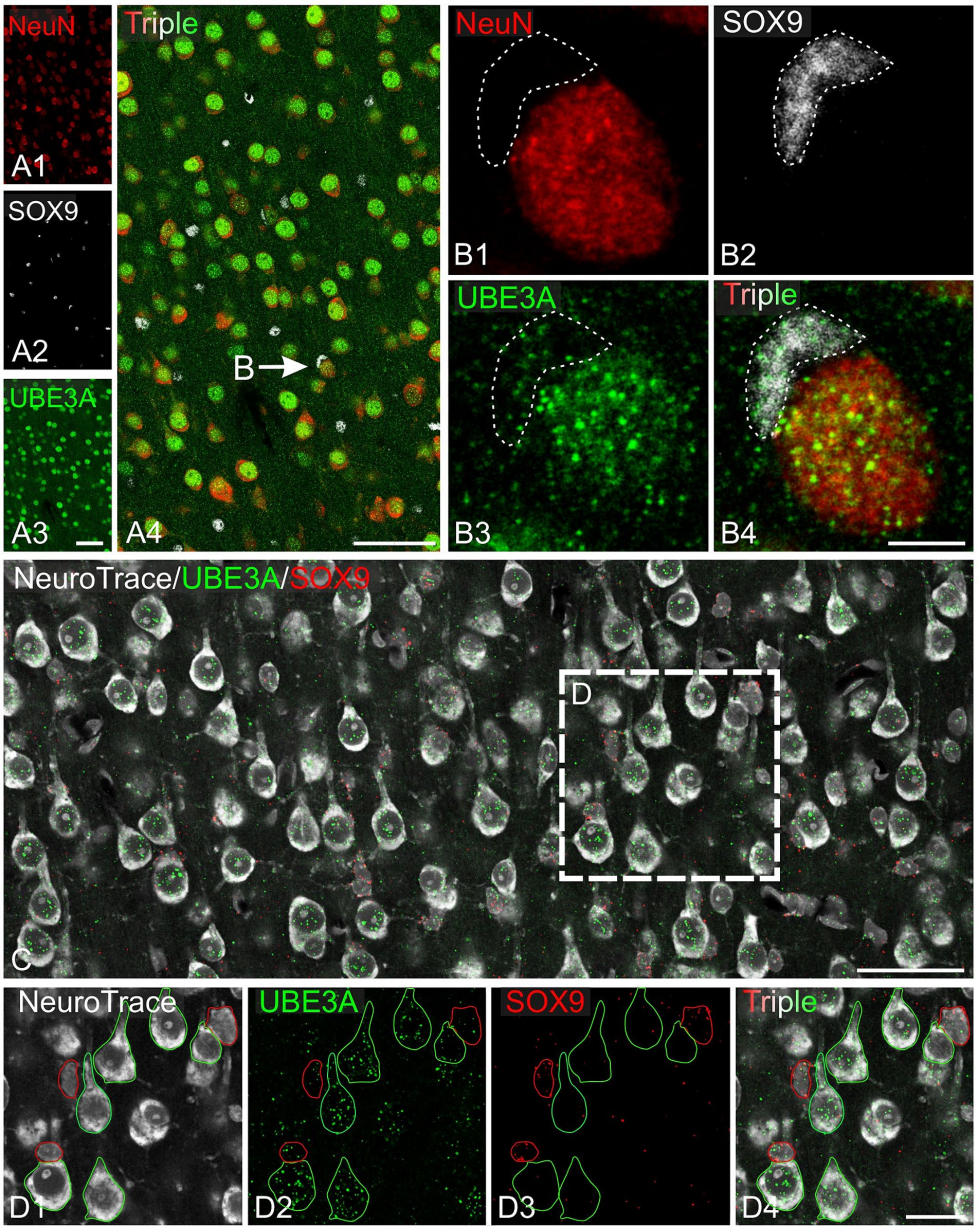
UBE3A expression in astrocytes in the one-month-old macaque brain. **(A)** Triple immunofluorescence labeling for UBE3A (green), the neuronal marker NeuN (red), and the glial marker SOX9 (white) in the somatosensory cortex. **(B)** Enlargement from **A** (arrow) shows a UBE3A-positive astrocyte (dashed area) adjacent to a neuron exhibiting stronger UBE3A expression. **(C)** Duplex detection of UBE3A (green) and SOX9 (red). **(D)** Enlargement of the boxed area from **C**. Scale bars: **A**: 50 μm, **B**: 5 μm; **C**: 50 μm, **D**: 25 μm.

**Figure 7:**
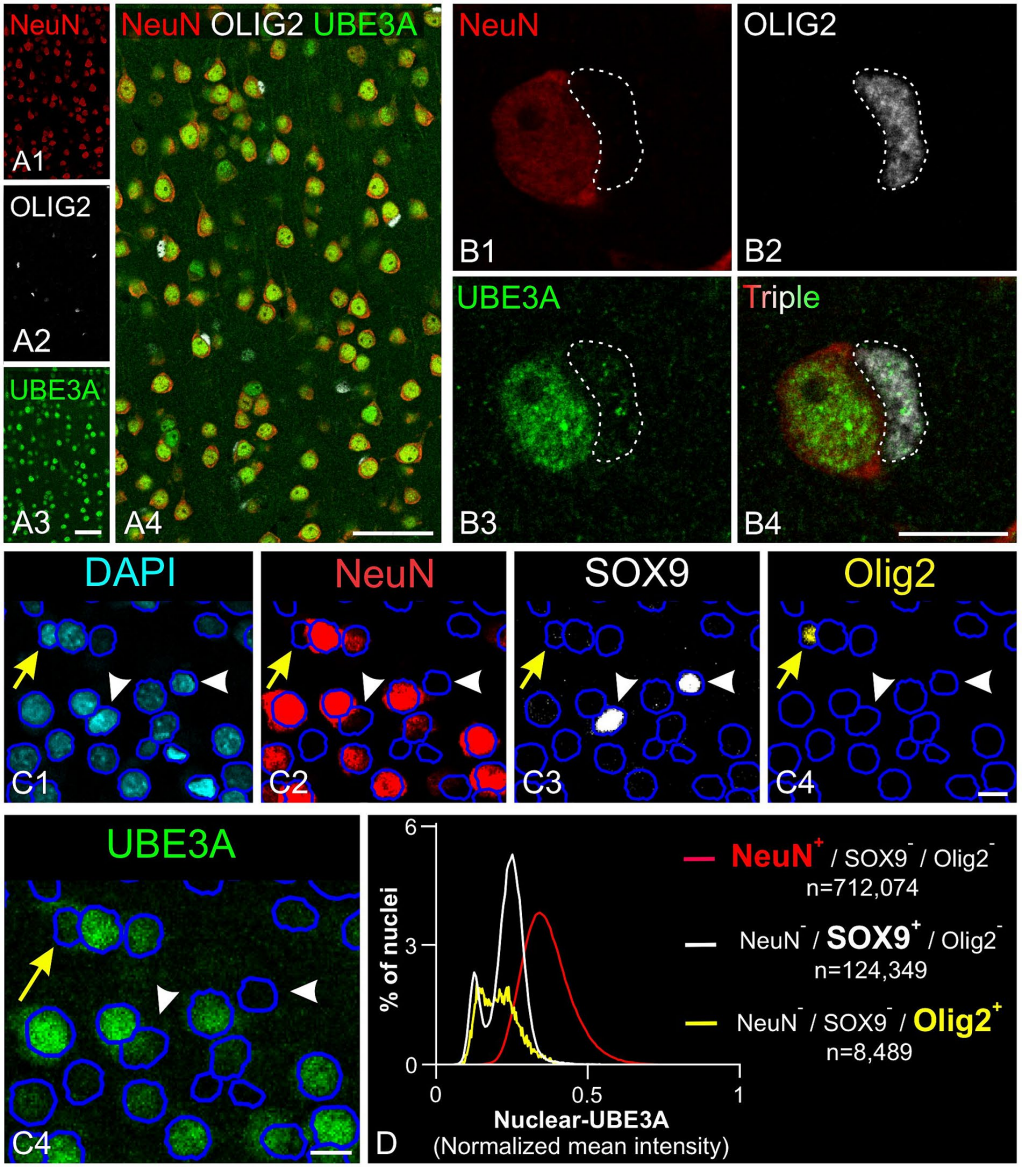
UBE3A expression in oligodendrocytes in the one-month-old macaque brain. **(A)** Triple immunofluorescence labeling for UBE3A (green), the neuronal marker NeuN (red), and the oligodendrocyte marker OLIG2 (white) in the somatosensory cortex. **(B)** A higher magnification image showing a UBE3A-positive oligodendrocyte (dashed area) next to a neuron with more intense UBE3A staining. **(C)** Quintuple labeling for UBE3A (green), the neuronal marker NeuN (red), the glial marker SOX9 (white), the oligodendrocyte market (yellow), and the nuclear marker DAPI (blue) with the accompanying deep learning nuclei segmentation (deep blue tracing) performed with StarDist. The yellow arrow points to a nucleus immunolabeled for Olig2 but not for NeuN or SOX9. White arrowheads point to nuclei immunolabeled for SOX9 but not for NeuN or OLIG2. **(D)** Histogram plot shows nuclear UBE3A fluorescence profiles in neurons (NeuN positive, but negative for SOX9 and OLIG2), astrocytes (SOX9 positive, but NeuN and OLIG2 negative), and oligodendrocytes (OLIG2 positive, but NeuN and SOX9 negative). Scale bars: **A**: 50 μm; **B**: 5 μm, **C**: 10 μm.

Next, we explored UBE3A expression in neurons and glial cells. Simultaneous labeling for UBE3A and the neuronal marker NeuN showed that nearly all NeuN-positive cells were also positive for UBE3A (Figure 5). However, the nuclear expression of UBE3A in neurons exhibited variability, as evidenced by heightened staining in larger nuclei and diminished levels in smaller nuclei (Figures 5B,C). Given the established connection between deficits in GABAergic circuitries and AS pathogenesis (Judson et al., 2016; Gu et al., 2019), we examined UBE3A expression in glutamatergic and GABAergic neurons. Multiplex ISH experiments indicated that the majority of VGLUT1-positive (glutamatergic) and VGAT-positive (GABAergic) neurons coexpressed UBE3A transcript (Figures 5D,G), and suggest lower UBE3A levels in VGAT-positive neurons (Figures 5E,F). Not all UBE3A-positive cells were identified as neurons based on NeuN, VGLUT, or VGAT expression, suggesting that a significant portion comprises glial cells. To confirm UBE3A expression in glial cells, we used multiple labeling and multiplex ISH for UBE3A and SOX9 (Figure 6). We chose to examine SOX9 over GFAP because SOX9 is known to be expressed in all astrocytes (Sun et al., 2017). Immunohistochemistry revealed UBE3A protein in all SOX9-positive nuclei, albeit at a lower level than neurons (Figures 6A,B). Complementary examination using *in situ* hybridization (ISH) shows astrocytes displayed reduced UBE3A mRNA labeling compared to neurons (Figures 6C,D). We also investigated UBE3A expression in oligodendrocytes, finding that, like astrocytes, this cell type appeared to have lower UBE3A protein levels than neurons (Figures 7A,B).

To further understand UBE3A expression variations among cell types, we compared UBE3A levels in neurons, astrocytes, and oligodendrocytes (Figures 7C,D). We selected the visual cortex for study because its structure and size facilitate the collection of small yet uniform pieces, thereby conserving the limited macaque tissue available. Moreover, changes in dendritic morphology have been observed within visual cortex in one of the only human AS anatomical studies to date (Jay et al., 1991), suggesting this is a potential site of pathology in Angelman and Dup15q syndromes. Cells were identified based on specific markers: NeuN for neurons, SOX9 for astrocytes, and Olig2 for oligodendrocytes. Neurons showed higher UBE3A expression compared to astrocytes and oligodendrocytes in agreement with our prior findings in mouse and human studies (Burette et al., 2017, 2018). Intriguingly, our data also suggest the existence of two distinct astrocyte populations based on UBE3A expression: a predominant group constituting 80% of all astrocytes and a smaller group (20%) characterized by approximately half the UBE3A concentration.

**Figure 8:**
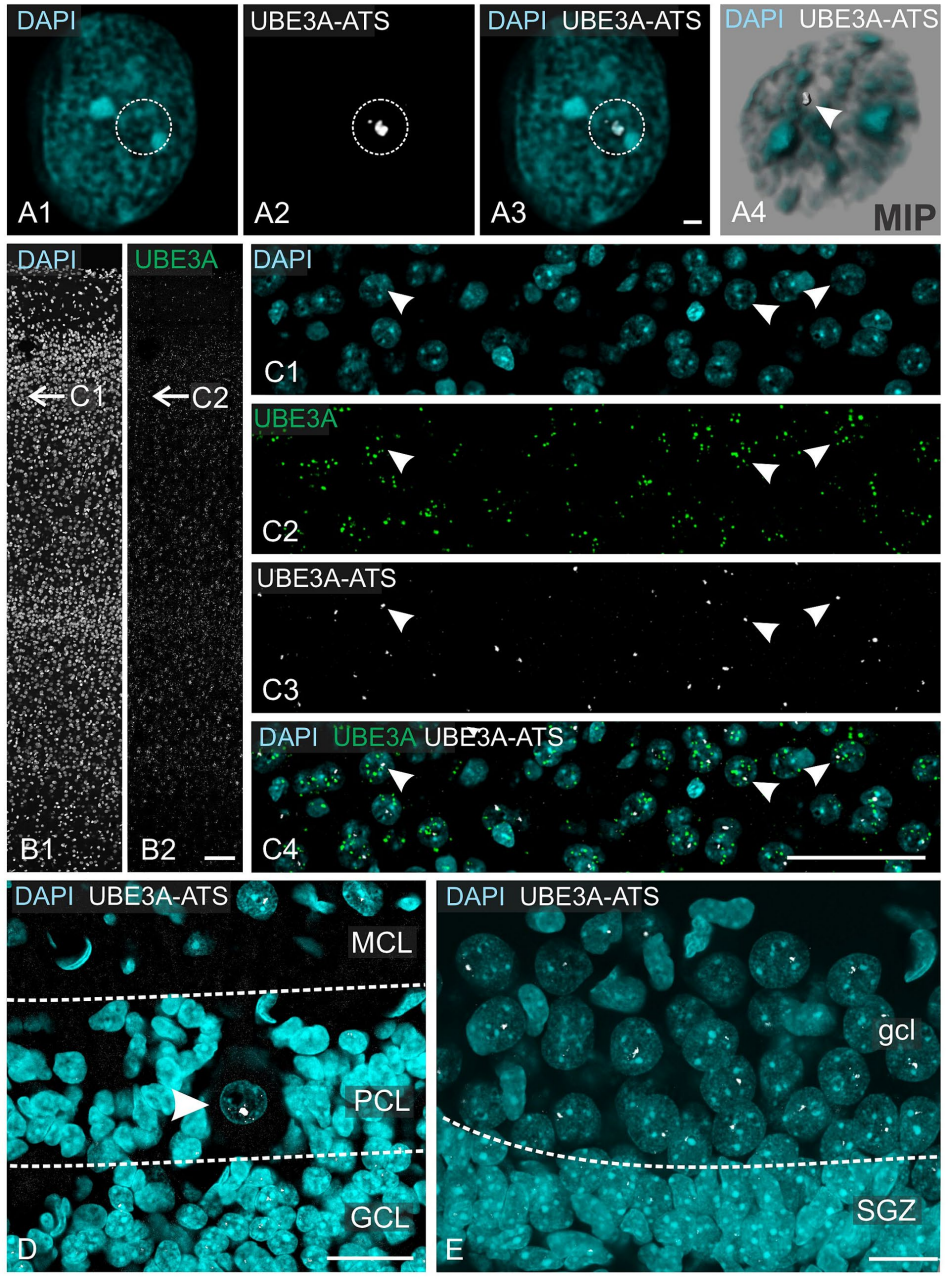
*UBE3A-ATS* expression in the one-month-old macaque brain. **(A1–A3)** *UBE3A-ATS* (white dotted circle) signal forms a single small nuclear bright dot. **(A4)** Maximum intensity projection of staining shown in **A1–A3**. **(B)** *In situ* hybridization detection of UBE3A in the somatosensory cortex. **(C)** Enlargement at the area marked by arrows in **B**. **(D)** *UBE3A-ATS* in the cerebellar cortex; note the expression of *UBE3A-ATS* in granule cells and the molecular cell layer, but its absence in probable radial glial cells. Arrowhead points to the nucleus of a Purkinje cell. **(E)** *UBE3A-ATS* in the dentate gyrus; note the expression of *UBE3A-ATS* in granule cells, but its absence in neuronal stem cells in the subgranular zone. GCL, granule cell layer (cerebellum); gcl, granule cell layer (hippocampus); MCL, molecular cell layer; PCL, Purkinje cell layer; SGZ, subgranular zone. Scale bars: **A**: 1 μm; **B**: 100 μm; **C**: 50 μm; **D**: 25 μm; **E**: 20 μm.

To gain insight into paternal *UBE3A* silencing, we performed ISH for *UBE3A-ATS* (Figure 8). In contrast to mRNAs, whose signals are scattered across the cytoplasm, the *UBE3A-ATS* signal was confined to nuclei and observed as small, bright focal puncta, often surrounded by several tiny speckles (Figure 8). A close 3D examination of the z-stack dataset showed a single *UBE3A-ATS* punctum per nucleus (Figure 8A). These observations are consistent with previous findings supporting that *UBE3A-ATS* expression is mono-allelic with a very short (~4 h) half-life (Meng et al., 2012).

**Figure 9:**
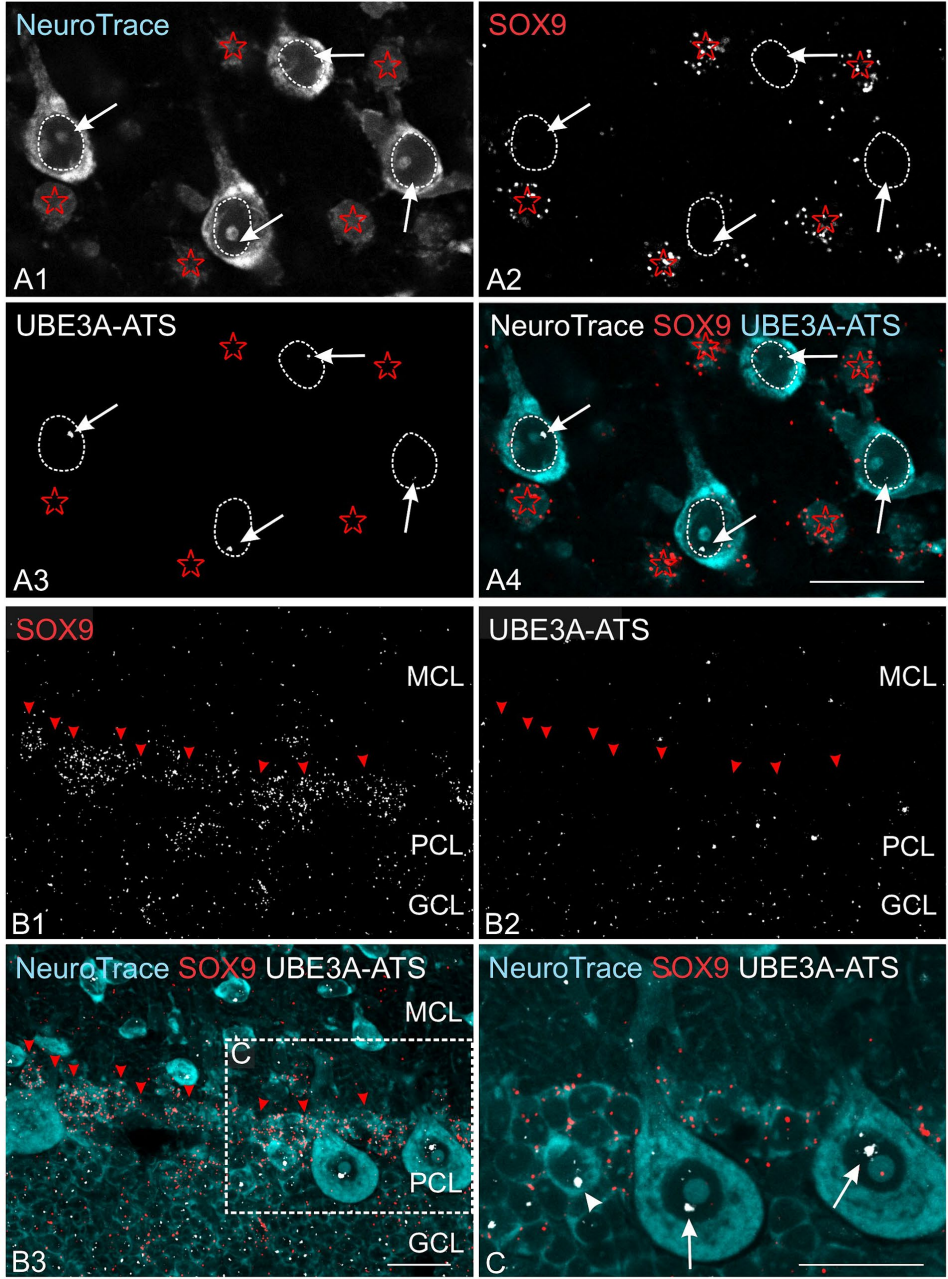
Astrocytes lack *UBE3A-ATS* expression in the one-month-old macaque. **(A)** Duplex detection of *UBE3A-ATS* and the astrocyte marker SOX9 in the somatosensory cortex. Note the absence of *UBE3A-ATS* signals in astrocytes, identified by their SOX9 expression (red asterisks). In contrast, *UBE3A-ATS* signals (white arrows) are detected in the neuronal nuclei outlined by white dotted lines. **(B)** Duplex detection of *UBE3A-ATS* and the astrocyte marker SOX9 in the cerebellar cortex. **(C)** Enlargement of the boxed area from **B**. In contrast to Purkinje cells (arrows), radial glial cells, identified by SOX9 signals in their cytoplasm, do not express *UBE3A-ATS*. Arrowhead points to probable Golgi cells expressing *UBE3A-ATS*. GCL, granule cell layer (cerebellum); MCL, molecular cell layer; PCL, Purkinje cell layer. Scale bars: 25 μm.

Multiplex experiments show the presence of UBE3A and *UBE3A-ATS* signals throughout the post-natal macaque brain (Figures 8B,C). However, not all UBE3A-expressing cells also exhibited a *UBE3A-ATS* signal. In areas characterized by well-organized anatomical structures, cells that lack *UBE3A-ATS* can be identified as non-neuronal based on their distinctive size, shape, and arrangement. For example, in the cerebellar cortex, *UBE3A-ATS* positive Purkinje cells nested between *UBE3A-ATS* negative Bergmann glia (Figure 8D). Of note, the subgranular zone of the hippocampal dentate gyrus, a prominent neurogenic niche in the adult brain, exhibited very little *UBE3A-ATS* signal (Figure 8E). Validation of the absence of *UBE3A-ATS* in glial cells was confirmed through multiplex ISH for UBE3A and SOX9 (Figure 9).

Collectively, these findings delineate a UBE3A expression pattern in the postnatal macaque brain closely resembling that observed in mice, characterized by robust nuclear expression in neurons across the whole brain and comparatively weaker expression in glial cells. Notably, the expression profile of *UBE3A-ATS* suggests that paternal *UBE3A* is silenced in neurons but not in glial cells, mirroring observations in mice.

**Figure 10:**
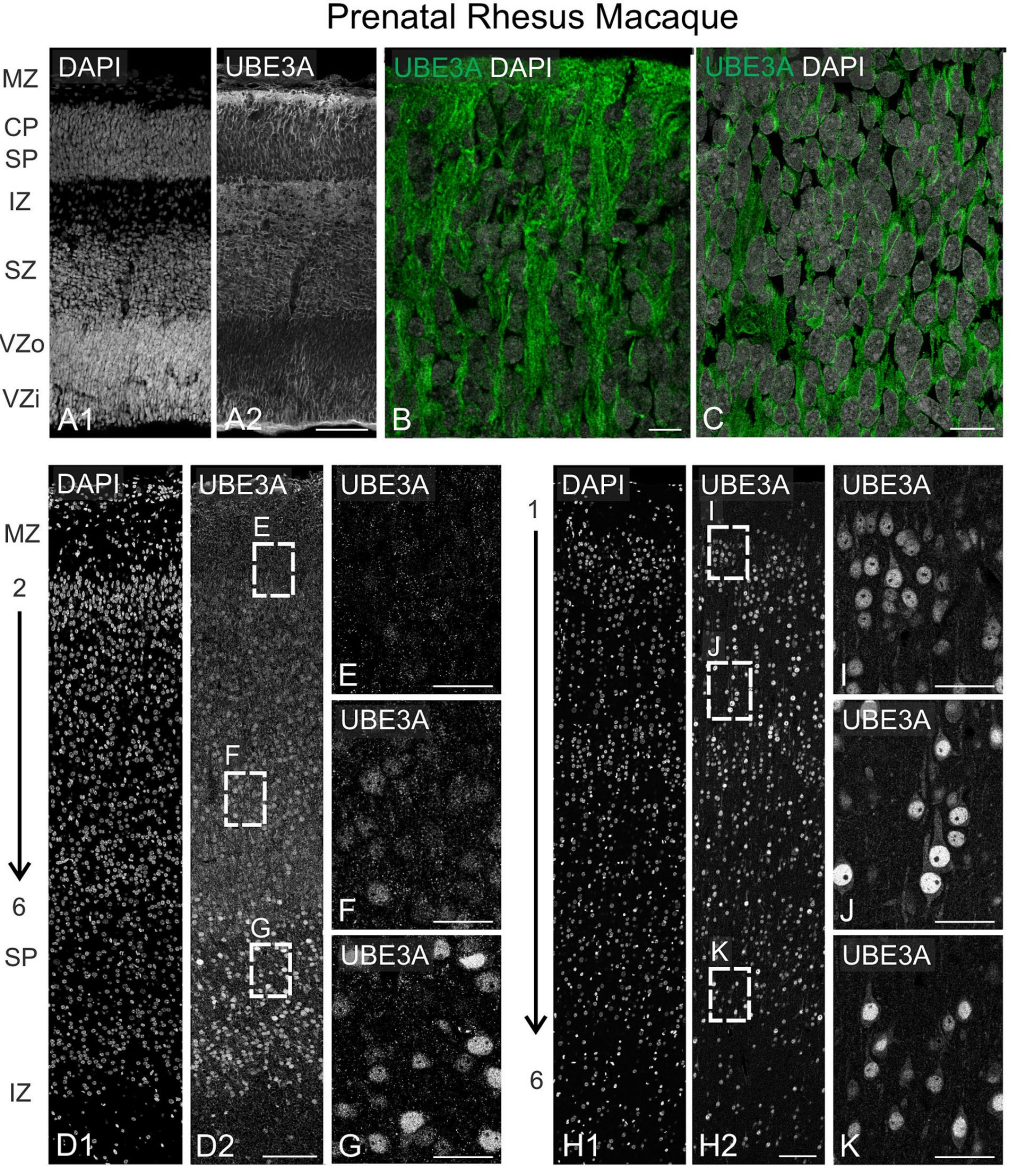
UBE3A expression in the embryonic macaque brain. **(A)** UBE3A (green) immunofluorescence co-stained with DAPI (grey) in the neocortex during neurogenesis of lower layer cortical neurons (GD 48). **(B)** Higher magnification of cortical plate/marginal zone. **(C)** Higher magnification of the ventricular zone. **(D)** UBE3A and DAPI staining in the neocortex during neurogenesis layer 2/3 neurons (GD 100). **(E–G)** Enlargement of the boxed areas from **D**. **(H)** UBE3A and DAPI staining in the GD 151 neocortex. **(I–K)** Higher magnification images from **H**. CP, cortical plate; IZ, intermediate zone; MZ, marginal zone; SP, subplate; SZ, subventricular zone; VZi, inner ventricular zone; VZo, outer ventricular zone. Scale bars: **A**: 100 μm; **B,C**: 10 μm; **D**: 100 μm; **E–G**: 25 μm; **H**: 100 μm; **I–K**: 25 μm.

**Figure 11:**
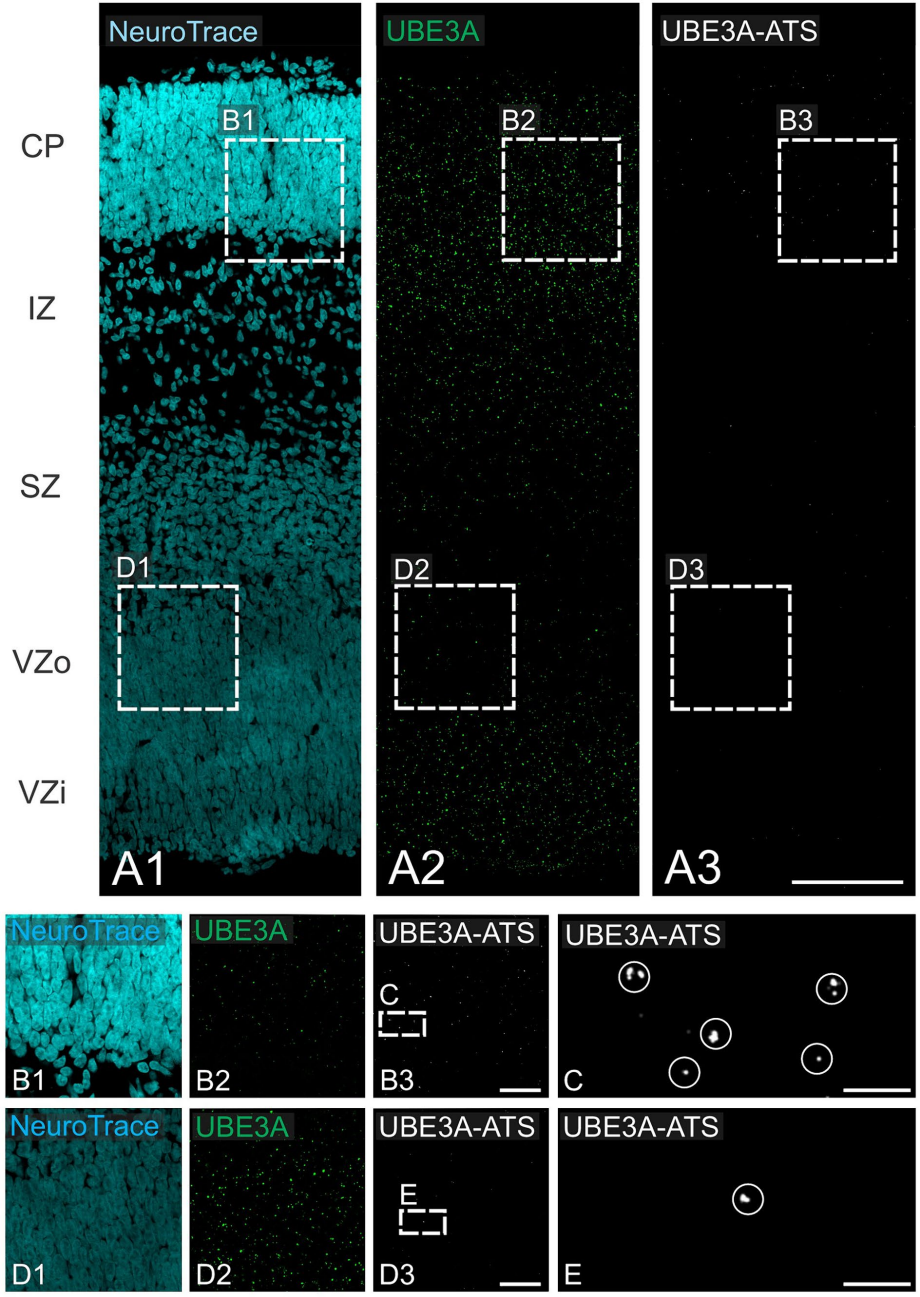
UBE3A and *UBE3A-ATS* expression in the neocortex of the macaque brain at GD 48. **(A)** Duplex detection of UBE3A (green) and *UBE3A-ATS* (white) during the neurogenesis of lower-layer cortical neurons (GD 48). **(B,D)** Enlargement of the boxed areas from **A**. **(C)** Enlargement of the boxed area from **B**, **(E)** Enlargement of the boxed area from **D**. Circles enclose *UBE3A-ATS* signal. CP, cortical plate; IZ, intermediate zone; SZ, subventricular zone; VZi, inner ventricular zone; VZo, outer ventricular zone. Scale bars: **A,B**: 100 μm; **C,E**: 25 μm; **D,F**: 5 μm.

To investigate UBE3A and *UBE3A-ATS* in the prenatal macaque brain, we focused on the neocortex. The neocortex has undergone significant expansion during primate evolution, lending to markedly greater complexity of this structure in the macaque compared to mice. Additionally, the well-studied ‘inside-out’ development of the neocortex provides the anatomical advantage of observing multiple developmental stages simultaneously across the cortical thickness. We studied three-time points: GD 48 during the neurogenesis of deep cortical layers, GD 100 during the neurogenesis of upper layers and the start of neuronal maturation in deep layers, and GD 151 toward the end of cortical synaptogenesis in all cortical layers (Wallace and Pollen, 2024). At GD 48, UBE3A staining was widespread across the cortical wall but most prominent at the interface of the marginal zone and the cortical plate and in the ventricular zone. Lower UBE3A intensity was evident in the subventricular zones (Figures 10A–C). At the subcellular level, UBE3A staining appeared diffuse, filling the thin layer of cytoplasm surrounding largely unstained nuclei and extending into cellular processes. In contrast to UBE3A’s widespread distribution, *UBE3A-ATS* signal was seldom observed at GD 48 (Figure 11).

**Figure 12:**
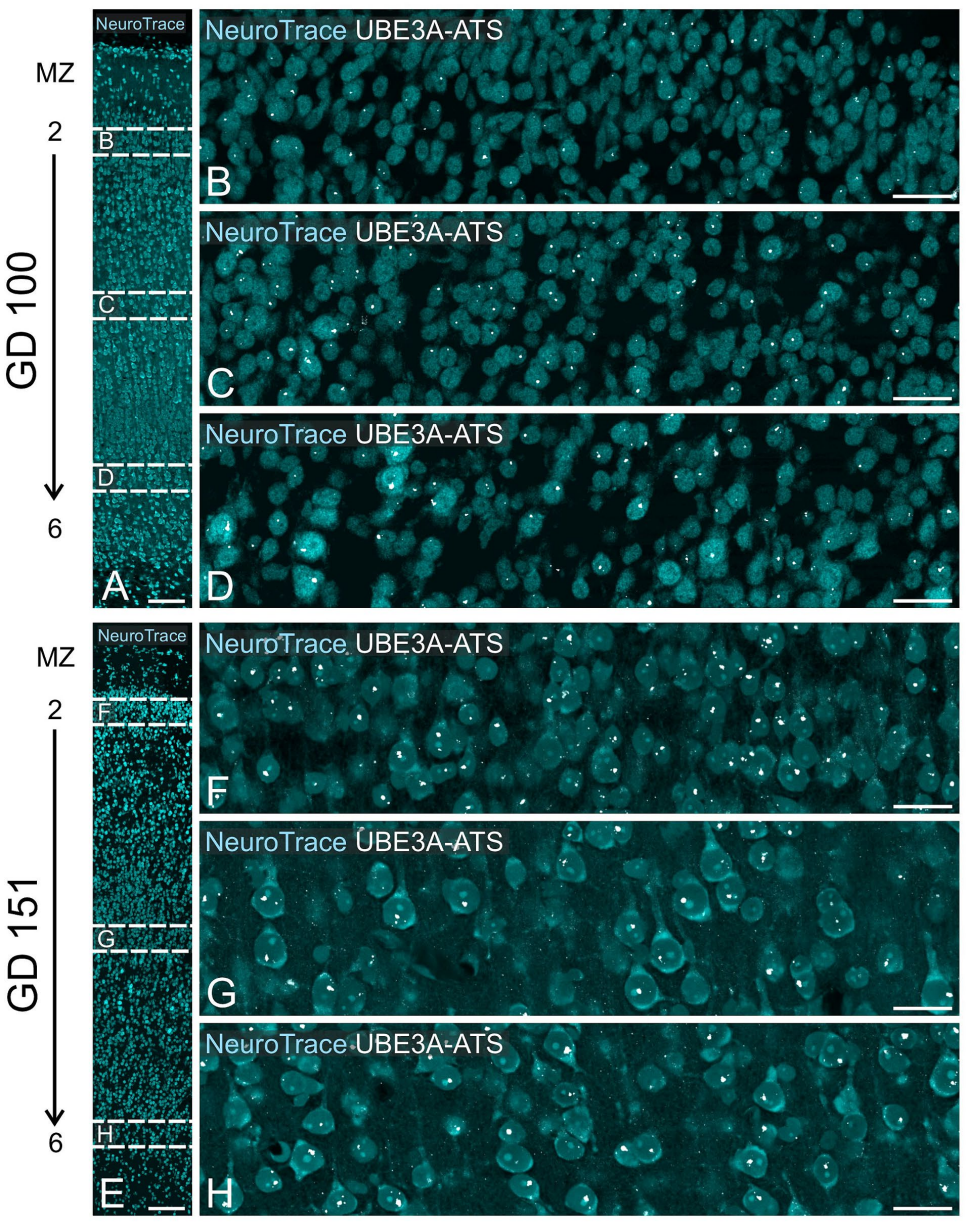
*UBE3A-ATS* expression in the neocortex of the macaque brain at GD 100 and GD 151. **(A)** GD 100 neocortex stained with NeuroTrace providing context for **B–D**. **(B–D)** Enlargement of the boxed area in **A** shows *UBE3A-ATS* signal counterstained with NeuroTrace. **(E)** GD 151 neocortex stained with NeuroTrace, providing context for **F–H**. **(F–H)** Enlargement of the boxed areas from **E** shows *UBE3A-ATS* expression counterstained with NeuroTrace. MZ, marginal zone. Scale bars: **A,E**: 100 μm; **B–D**, **F–H**: 25 μm.

At GD 100, there were significant changes in UBE3A subcellular localization (Figures 10D–G). In the superficial layers, UBE3A staining remained predominantly extra-nuclear, like at GD 48. However, in the deeper, more developed layers, UBE3A was primarily located inside the nucleus, as it is after birth. This developmental shift in UBE3A subcellular distribution was accompanied by changes in *UBE3A-ATS* expression, with cells in the more mature layer 6 exhibiting a notable increase in *UBE3A-ATS* compared to their younger counterparts near the overlying marginal zone (Figures 12A–D). At GD 151, UBE3A staining was present throughout the developing cerebral cortex, resembling the postnatal distribution (Figures 10H–K). UBE3A staining concentrated in cell nuclei across all layers, with additional staining in the cytoplasm, cellular processes, and neuropil, albeit at reduced levels. *UBE3A-ATS* signal was found in numerous cells, many displaying sufficient maturity to be identified as neurons when counterstained with NeuroTrace (Figures 12E–H).

Our findings indicate a dynamic shift in UBE3A subcellular localization between GD 48 and GD 151, with UBE3A progressively becoming more nuclear. Interestingly, this shift coincides with the increase in *UBE3A-ATS* expression in mature neurons.

**Figure 13:**
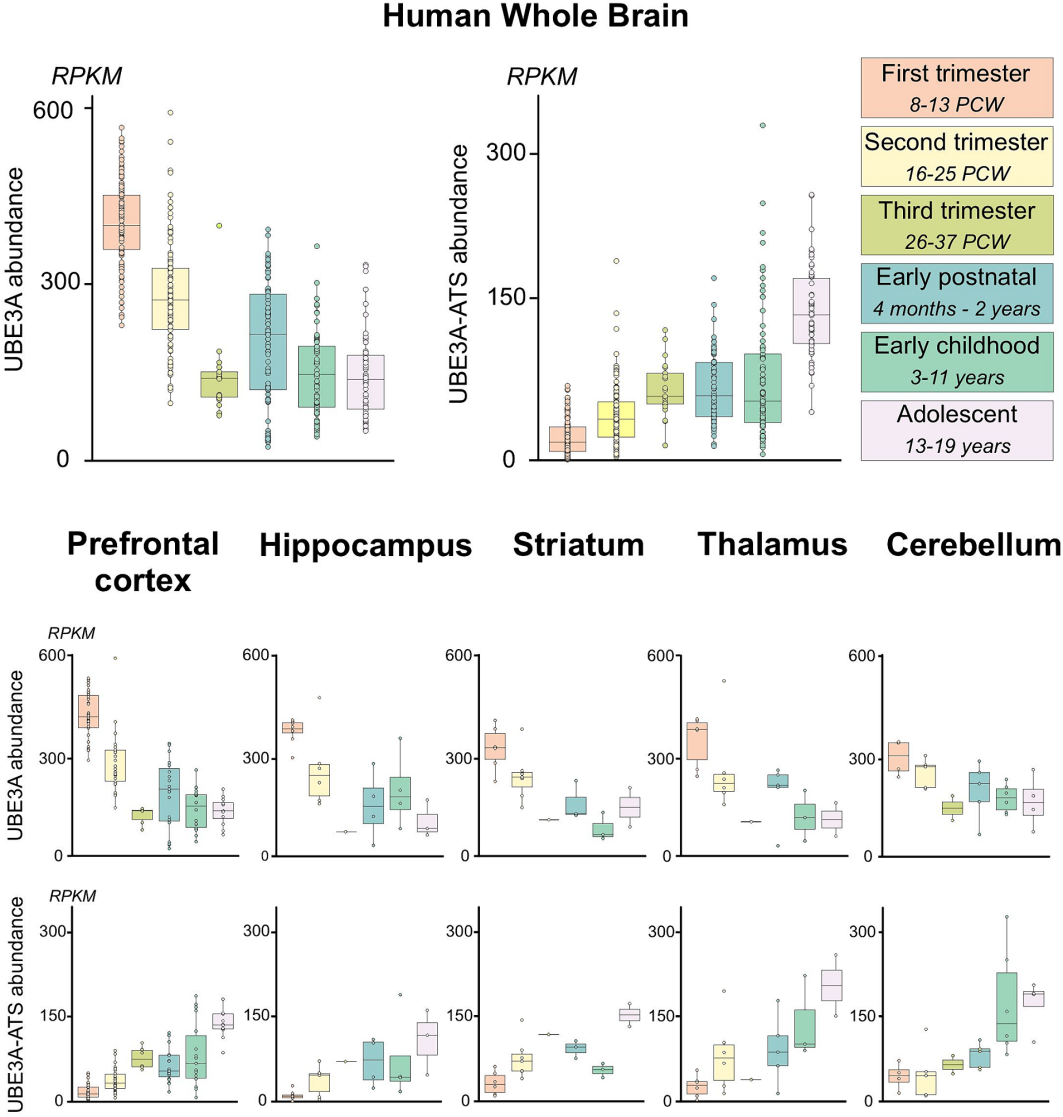
hUBE3A and *UBE3A-ATS* expression in the human brain across development from publicly available human RNA sequencing data. Boxplots of *human UBE3A* and *UBE3A-ATS* expression in human brain specimens across developmental time. Data were obtained from BrainSpan (http://www.brainspan.org/static/download.html). *UBE3A-ATS* abundance was estimated using expression of exons within the neuron-specific portion of the *UBE3A-ATS* (chr15:25400000–25550000; hg19/GRCh37) as described by Hsiao et al. (2019). Expression was summarized by brain regions and developmental epochs as previously described (Judson et al., 2021). Upper panel: human *UBE3A* and *UBE3A-ATS* expression in the developing human brain. Lower panel: human *UBE3A* and *UBE3A-ATS* expression across multiple regions of the developing human brain.

While the overall trends in the spatial and temporal gene expression patterns are generally similar between humans and macaques, variations exist. These differences are particularly evident in genes related to synapse formation, neuronal development, and myelination (Zhu et al., 2018). The limited availability of high-quality human tissue samples has hindered our ability to perform detailed histological analyses. Therefore, to determine the applicability of our findings to humans, we analyzed publicly available human RNA sequencing data (Figure 13). *UBE3A* expression peaked during the first trimester in humans, after which it gradually decreased until birth. Following a brief increase in the early postnatal period, the expression levels of *UBE3A* stabilize after three years of age, aligning with levels seen at birth. In contrast, the expression of *UBE3A-ATS* is very low during the first trimester but steadily rises until adolescence. When examined in specific brain regions, these expression patterns show similar trajectories, with minor variations in timing that may be attributed to differences in developmental progression. Notwithstanding the developmental asynchrony observed between macaques and humans, these data suggest that the dynamic expression profiles of UBE3A and *UBE3A-ATS* in humans closely agree with our histological observations in macaques.

## 4 Discussion

Mice have been the foundation for understanding UBE3A biology. Mouse models have played a critical role in characterizing AS and Dup15q syndrome pathology and are extensively used to design and screen therapeutic strategies. For example, AS mouse models successfully recapitulate various aspects of AS, including seizures and motor deficits. However, these mouse models cannot replicate the full spectrum of phenotypes, especially those related to severe intellectual disability, language impairment, and complex social behaviors. Therefore, as with other disorders affecting core human traits, a key challenge is determining the extent to which findings in murine models can be effectively translated to individuals with AS and Dup15q syndromes. This challenge arises from the substantial evolutionary divergence (~80 million years) between mice and humans, leading to significant discrepancies across various biological domains. These discrepancies range from the genomic and cellular level to neurocircuitry and complex behaviors (Izpisua Belmonte et al., 2015; Kaiser and Feng, 2015; Jennings et al., 2016). Transcriptomic analyses highlight these limitations by revealing substantial differences in molecular signatures between mice and primates; notably, genes implicated in neurological disorders display these variations (Parikshak et al., 2015; Pembroke et al., 2021). Studying UBE3A in the macaque brain thus provides a crucial complement to mouse studies and helps to bridge the gap between rodent and human research. Here, we provide the first detailed account of UBE3A and *UBE3A-ATS* expression in developing primate brains.

Despite species-specific variations in developmental timing and brain anatomy, our findings reveal remarkable conservation in the developmental trajectory of UBE3A expression between macaques and mice. This strengthens the validity of mouse models for investigating UBE3A-related disorders. Key similarities include a broad regional distribution across all ages examined, expression in most brain cell types, with consistently higher levels in neurons (both excitatory and inhibitory) compared to non-neuronal glial cells, and, most obvious in neurons, a developmental shift from cytoplasmic to predominantly nuclear subcellular localization. Importantly, the expression pattern of *UBE3A-ATS* in macaques suggests, akin to mice, that paternal *UBE3A* is developmentally silent in neurons and not in glial cells. The conservation of critical UBE3A biological features between the two species suggests the preservation of functionality. For example, the subcellular location of UBE3A likely influences its interaction with different substrates and, consequently, its functional activity. In particular, the noted similarity in UBE3A’s developmental transition from the cytoplasm to the nucleus in both mice and macaques suggests that UBE3A’s cellular functions are highly conserved during neuronal maturation.

**Figure 14:**
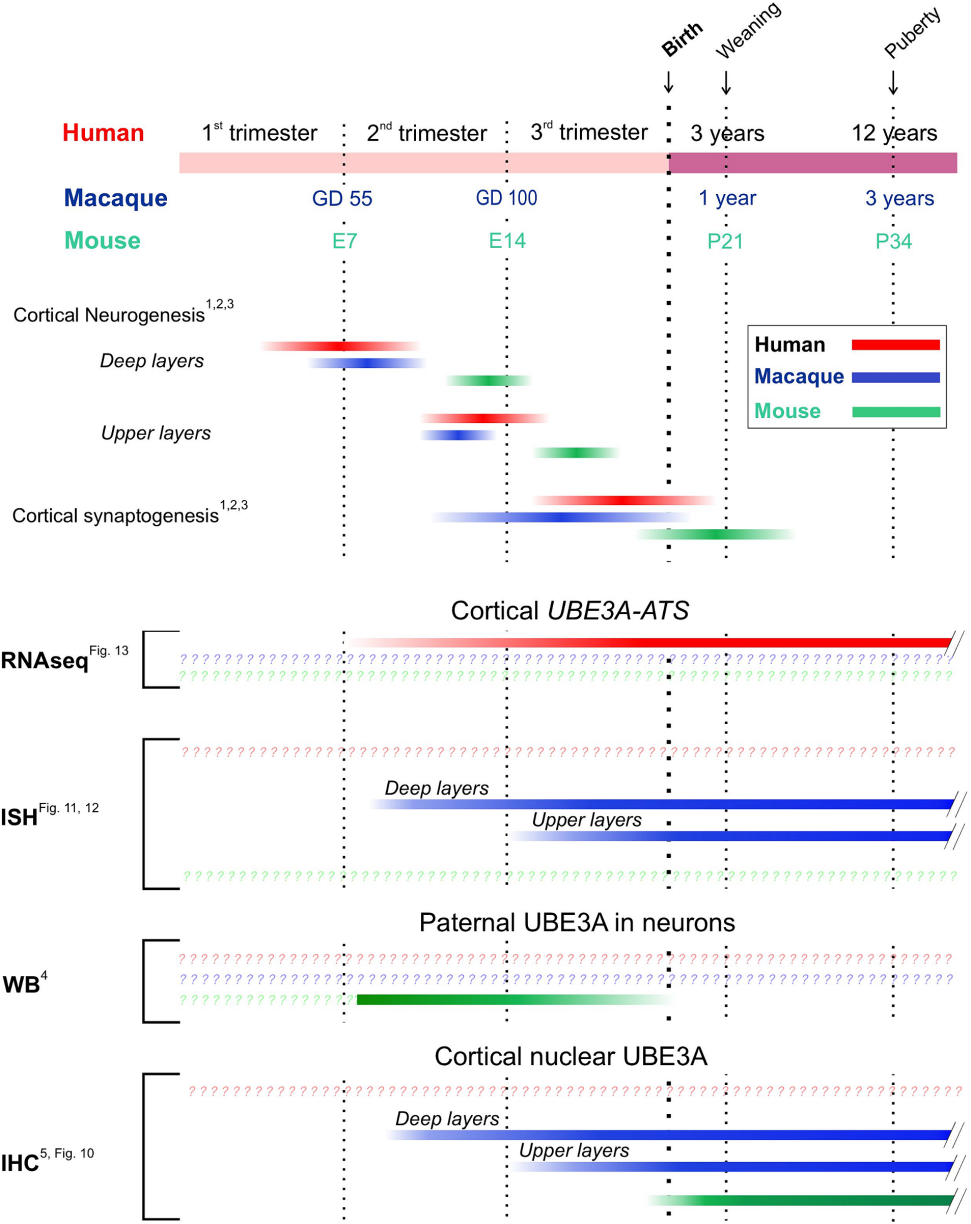
UBE3A developmental biology in the neocortex across species. Upper panel: timings of key developmental milestones in the mouse, rhesus macaque, and human brain adapted from Wallace and Pollen (2024) and references within. Lower panel: timings of key developmental milestones of UBE3A biology. ^1^Wallace and Pollen (2024), ^2^van den Ameele et al. (2014), ^3^Rakic (1975), ^4^Sonzogni et al. (2020), and ^5^Judson et al. (2014).

Translating developmental data from animal models to humans presents challenges due to complex timing differences across species (Wallace and Pollen, 2024). Determining exact equivalencies is difficult, as factors like cerebral growth, neurogenesis, and synaptogenesis vary. This complexity is further compounded by regional differences in brain development timing. Despite this, our developmental data offer valuable insights, especially when focusing on neocortical development (Figure 14).

In the neocortex, neuron formation progresses from the inside out. Neurons destined for the outer layers migrate past those forming the deeper layers. Consequently, neurons in the deeper layers mature days before those in the upper layers. In mice, neurogenesis begins in the deeper layers around E11 and finishes in the upper layers by E18 (van den Ameele et al., 2014; Kroon et al., 2019). This is followed by a rapid maturation period for principal neurons during the first two postnatal weeks. The cytoplasmic-to-nuclear shift of UBE3A also occurs during this maturation phase (Judson et al., 2014). The precise profile of *Ube3a-ATS* expression in mice is unclear. Although *Ube3a-ATS* can be detected as early as E10 in mice (Meng et al., 2013), analyses of cortical lysates via Western blot revealed that the paternal *Ube3a* allele is fully silenced in neurons soon after birth (Sonzogni et al., 2020), following the completion of neurogenesis, within the neuronal maturation period. This developmental epoch in mice is very brief, making extrapolation to human timelines difficult. Macaque brain development, similar to human, is much more protracted, leading to a greater temporal resolution of key aspects of UBE3A and *UBE3A-ATS* biology. We show that in macaques, the increase in *UBE3A-ATS* expression and the shift of UBE3A to the nucleus happen after neurogenesis, during the initial phases of neuron maturation, and before synapse formation (Rakic, 1975; Wallace and Pollen, 2024). Specifically, in the deeper layers of the cortex, the increase in *UBE3A-ATS* expression begins between GD 48 and GD 100, while in the outer layers, this increase starts around 50 days later, between GD 100 and GD 151. Therefore, our data support a conserved timeline between mice and macaques where maternal *UBE3A* silencing and nuclear localization happen post-neurogenesis, coinciding with neuronal maturation.

Fewer data are available for the developing human brain. Our RNA-Seq analysis indicates that in the prefrontal cortex, *UBE3A-ATS* expression begins in the second trimester and peaks in the third, suggesting that, as in mice and macaques, the maternal allele silencing of *UBE3A* in the human brain also takes place after the completion of neurogenesis and neuronal migration, as principal neurons begin to mature. Understanding the timeframe when *UBE3A-ATS* expression begins to silence paternal *UBE3A* is crucial. Before this period, expression of *UBE3A* from the paternal allele may partially offset the maternal allele loss in neurons. Once the paternal allele is fully silenced, however, maternal *UBE3A* loss is likely to be much more impactful. Thus, restoring UBE3A function might yield the greatest long-term benefits if done before closure of this window, which our data indicate lies between the late second and mid-third trimesters. This proposed timing is supported by mouse studies, where the critical window for reversing most behavioral phenotypes spans from birth to P21, the period when neuronal maturation occurs (Silva-Santos et al., 2015; Sonzogni et al., 2020; Milazzo et al., 2021). After P21 in mice, UBE3A functional reinstatement can still mitigate specific conditions such as susceptibility to seizures and alterations in sleep patterns, suggesting that even late reinstatement of UBE3A expression can provide clinical benefits despite having a narrower impact (Rotaru et al., 2018; Gu et al., 2019; Spencer et al., 2022; Lee et al., 2023).

Collectively, our findings support the validity of mouse models for developing and testing AS therapies. Furthermore, they suggest the middle third of pregnancy as an ideal window in humans, during which restoring UBE3A function could yield the greatest benefits. While these findings are promising, they highlight rather than detract from the need for an AS model in macaques. The spatiotemporal expression of UBE3A is crucial for its function, yet this is just one element influencing UBE3A’s biology. Such a model would not only enhance our comprehension of AS, but would also improve the evaluation of both the effectiveness and, critically, the safety of potential treatments during early development, offering a model with greater predictive accuracy for human outcomes.

## Data availability statement

The raw data supporting the conclusions of this article will be made available by the authors, without undue reservation.

## Ethics statement

All macaque monkey experimental procedures were approved by the Institutional Animal Care and Use Committee of the University of California, Davis. The study was conducted in accordance with the local legislation and institutional requirements.

## Author contributions

CGR: Investigation, Methodology, Writing – review & editing. SGS: Investigation, Methodology, Writing – review & editing. RKRP: Investigation, Methodology, Writing – review & editing. SC: Investigation, Methodology, Writing – review & editing. NWM: Methodology, Writing – review & editing. LMJ: Methodology, Writing – review & editing. NWR: Methodology, Writing – review & editing. JMS: Formal analysis, Methodology, Writing – review & editing. JB: Resources, Writing – review & editing. DGA: Methodology, Resources, Writing – review & editing. ACB: Conceptualization, Data curation, Formal analysis, Investigation, Methodology, Supervision, Visualization, Writing – original draft, Writing – review & editing. BDP: Funding acquisition, Writing – review & editing, Conceptualization, Methodology, Supervision.

## Glossary

**Table:** 

AS	Angelman syndrome
CA	Cornu ammonis
CP	Cortical plate
DG	Dentate gyrus
DMSO	Dimethylsulfoxide
GCL	Granule cell layer
GCL	Granule cell layer (cerebellum)
gcl	Granule cell layer (hippocampus)
HECT	Homologous to E6-AP carboxyl-terminus
ISH	*In situ* hybridization
ITG	Inferior temporal gyrus
IZ	Intermediate zone
LG	Lateral geniculate nucleus
MCL	Molecular cell layer
MTG	Middle temporal gyrus
MZ	Marginal zone
PBS	Phosphate-buffered saline
PCgG	Posterior cingulate gyrus
PCL	Purkinje cell layer
PCW	Postconceptional weeks
PoG	Postcentral gyrus
PrG	Precentral gyrus
RPKM	Kilobase per million reads
SGZ	Subgranular zone
SMG	Supramarginal gyrus
SP	Subplate
STG	Superior temporal gyrus
Sub	Subiculum
SZ	Subventricular zone
Th	Thalamus
UBE3A	Ubiquitin protein ligase E3A
VZi	Inner ventricular zone
VZo	Outer ventricular zone
WM	White matter

## Footnotes

^1^ http://www.brainspan.org/static/download.html
